# Label-free quantitative proteomic analysis of functional changes of goat milk whey proteins subject to heat treatments of ultra-high-temperature and the common low-temperature

**DOI:** 10.1016/j.fochx.2024.101691

**Published:** 2024-07-23

**Authors:** Majida Al-Wraikat, Mohamed Aamer Abubaker, Yingli Liu, Xi Ping Shen, Yu He, Linqiang Li, YongFeng Liu

**Affiliations:** a College of Food Engineering and Nutritional Science, Shaanxi Normal University, Xi'an, 710119, China; b Hospital of Shaanxi Normal University, Shaanxi Normal University, Xi'an 710119, China.

**Keywords:** Goat milk, Whey protein, Heat treatments, Label-free, Quantitative proteomics

## Abstract

This work investigated the functional changes in whey proteins obtained from goat milk subject to various temperature treatments. Ultra-high temperature instantaneous sterilization (UHTIS) caused less damage than the common low-temperature, whereas spray-drying treatment had the opposite effect. A total of 426 proteins were identified in UHTIS and control treatment groups, including 386 common proteins and 16 and 14 unique proteins. The UHTIS treatment upregulated 55 whey proteins while down-regulated 98. The UHTIS-treated whey proteins may upregulate three metabolic pathways but downregulate one. Overall, UHTIS only slightly impacted the composition and functions of whey proteins from goat milk compared to the common low-temperature treatments.

## Highlights

• Ultra-high temperature instantaneous sterilization (UHTIS) of milk was investigated.
• UHTIS caused less damage to goat whey proteins than low-temperature treatment.
• Spray-drying treatment had a more damaging effect on goat whey proteins.
• UHTIS upregulated 55 whey proteins and down-regulated 98 in goat milk.
• UHTIS slightly impacted the composition and functions of goat whey proteins.

## 1 Introduction

Heat treatment is an essential process to ensure the safety of milk products and is broadly divided into low-temperature and high-temperature methods (Dash et al., 2022). Low-temperature treatments (below 100 °C), commonly used to produce pasteurized milk, are traditionally believed to preserve the best nutritional and flavor qualities (Liu et al., 2020). In contrast, high-temperature treatments (above 100 °C), such as ultra-high temperature (UHT, 135–150 °C) and spray-drying temperature (SDT, above 150 °C), are more commonly employed in milk processing. These methods can eliminate virtually all microorganisms, including heat-resistant bacteria and spores in milk, thereby significantly extending the shelf-life of dairy products. However, such heat treatment can induce various changes in the physicochemical properties of milk constituents (Liao, Liu, & Ku, 2017; Moatsou, 2023), including the denaturation of proteins, interactions between the denatured proteins and the casein micelles, non-enzymatic browning, and the conversions of soluble calcium, magnesium and phosphate to the colloidal state (Tarhan & Kaya, 2021). These alterations can reduce the milk's nutritional and functional qualities. Despite these impacts, few studies have focused on the composition and function of whey proteins through high-temperature treatments.

Milk mainly contains two types of proteins: casein (80%) and whey protein (20%) (Tarhan & Kaya, 2021; Goulding, Fox, & O'Mahony, 2020). Casein is a heat-stable protein group that does not aggregate during or after heating; in contrast, whey protein, which includes active components like *β-lactoglobulin*, *α-lactalbumin*, and *immunoglobulins*, is heat-sensitive (Donato & Fanny Guyomarc'h., 2009). Upon exposure to high temperatures, whey proteins irreversibly denature and coagulate. Although whey proteins comprise a smaller proportion of milk proteins, they are crucial in enhancing immune functions, promoting gut health, and possessing insulin -otropic and glucose-lowering properties (Čurlej et al., 2022). Furthermore, the peptides and amino acids generated from the digestion of whey protein in the gastrointestinal tract stimulated the release of several gut hormones, such as cholecystokinin, peptide Y·Y, the incretins gastric inhibitory peptide, and glucagon-like peptide one that potentiate the secretion of insulin from β-cells associated with regulation of food intake (Duca, Waise, Peppler, & Lam, 2021). Despite the prevalence of high-temperature processed milk products like UHT milk and milk powder, there is a gap in research regarding the thermal stability of whey proteins derived from milk, especially goat milk (Ali et al., 2021). Like human breast milk, goat milk is highly regarded for its nutritional quality and digestibility.

This research investigates the changes in composition and physiological functions of whey proteins from goat milk subject to the common low-temperature, UHT, and SDT treatments. By employing a label-free quantitative proteomic approach, the study characterizes the profile changes in whey proteins and evaluates the potential functionalities of these significantly altered proteins. This could inform strategies to develop UHT goat milk whey protein products that retain more active constituents, contributing to the broader goal of maximizing the nutritional benefits of dairy products (Saadi et al., 2024).

## 2 Materials and methods

### 2.1 Preparation of whey protein from goat milk

The milk of Saanen goats (Weinan City, Shaanxi Province, China), selected as the experimental milk, contained 3.6% protein, 5.8% fat, 4.6% lactose, and 0.86% minerals. The goat milk was collected and delivered to the laboratory in sterile glass containers with ice packs. The cream of the fresh goat milk was removed through centrifuging (5,000*g* at 2 °C for 20 min). The chymosin was added to the skimmed goat milk by 1 g/L to form the curd. The curd was cut into small pieces to release whey, and the whey was ultimately obtained through centrifuging (8, 000*g* at 2 °C for 30 min). The whey was randomly divided into nine equal parts, with every three parts forming a group. One group was heated at 135 °C (UHT) for 6 s, another treated at 160 °C (SDT) for 6 s, and the untreated group served as the control. All the whey samples were placed on ice and added in 1 mL of protease inhibitors (50 mmol/L, DL-dithiothreitol, DTT), followed by centrifuging under 12,000*g* at 4 °C for 30 min to obtain the whey protein supernatants (Rodriguez-Ochoa, Cortes-Reynosa, Rodriguez-Rojas, de la Garza, & Salazar, 2023; Saadi et al., 2024).

### 2.2 Sodium dodecyl sulfate- polyacrylamide gel electrophoresis (SDS-PAGE)

SDS-PAGE was performed in a standard vertical gel electrophoretic apparatus, model SE-400 (Hoeffer Sci. Inst., San Francisco, CA, USA), according to Saliha, Dalila, Chahra, Saliha, and Abderrahmane (2013), the procedure described earlier with some modifications 5 mL of sample buffer was prepared with 1.25 mL of Tris-HCl of pH 6.8, 0.25 mL of β-mercapto ethanol, 0.5 g of SDS, 25 mg of bromophenol blue (BPB), and 2.5 mL of glycerol. Samples were dissolved into the sample buffer. After heating at 100 °C for 30 min, 20 μL of sample solution was loaded into the gel. The SDS-PAGE was carried out in the stacking gel under voltage 80 V and the separation gel under voltage 120 V. The gel was stained with R-250 Coomassie blue solution (2.5 g Coomassie Brilliant Blue R-250 was dissolved in the mix of 450 mL of methanol, 450 mL of water, and 100 mL of glacial acetic acid) for 2 h, followed by overnight destaining in a solution containing 25% (*v*/v) methanol and 10% (v/v) acetic acid.

### 2.3 Measurements of diameter and morphology of whey protein particles

The diameter and morphology of whey protein particles were measured using the method of Kodera, Yamamoto, Ishikawa, and Ando (2010) and MultiMode8 atomic force microscopy (Bruker Corporation, Billerica, Massachusetts, U.S.). The heated whey protein solutions were diluted 25 times with distilled water, dropped on the mica sheet naturally dried and measured with a J probe.

### 2.4 Determination of whey protein concentration

The concentration of whey protein was estimated according to the bicinchoninic acid (BCA) method described by Sun et al. (2018) and using the BCA kit (Thermo et al., U.S).

A series of concentrations (0, 0.125, 0.25, 0.5, 0.75, 1, 1.5, and 2 μg/μL) of bovine serum albumin (BSA) was prepared, and the absorbance values of BSA solutions were determined using a microplate reader (SpectraMax 190, Molecular Devices, U·S) at 562 nm. A standard curve was established by plotting the absorbance value against the corresponding concentration (y = 0.745× + 0.009, R2 = 0.995; y and x indicate the absorbance value and the concentration, respectively. The correlation coefficients were used to measure the credibility of the linear relationship between the absorbance value and the concentration.

### 2.5 LC-MS/MS analysis

According to the manufacturer's protocol, whey protein was purified using HIS–Select Nickel affinity gel (Sigma-Aldrich, St. Louis., MO, USA). Then, LC-MS/MS analysis was performed in the following steps.

Step1: Whey protein reduction alkylation and enzymatic hydrolysis. Tris (2-carboxymethyl) phosphate hydrochloride (TECP) was added to 10 mg of the whey protein supernatant. Subsequently, iodoacetamide (IAM) was added and oscillated at room temperature in the dark for 40 min to conduct the reductive alkylation reaction. Precooled acetone was added by the ratio 6:1 of acetone to sample volume to each tube to precipitate whey protein at −20 °C, centrifuged at 10,000*g* for 20 min to get the sediment of the whey protein*,* dissolved thoroughly with triethyl-ammonium bicarbonate buffer (TEAB). Trypsin was added by a mass ratio of 1:40 (m/m) to digest protein overnight at 37 °C.

Step 2: Peptide segment desalination and quantification. The peptide segments were desalinated by C18, dried with a vacuum pump, and dissolved with TEAB to a final concentration of 0.25 μg/μL, determined using a peptide quantification kit (Thermo Scientific, USA).

Step 3: Liquid chromatography-tandem mass spectrometer (LC-MS/MS). The desalinated peptide segments were separated using a liquid chromatography (LC) system with a temperature-controlled autosampler and column compartment (EASY-nLC 1, 200, Thermo Scientific, USA). 20 μL of peptide mixtures were loaded on Acclaim PepMap100 C18 nano-trap column (75 μm × 75 mm, 3 μm, 100 Å; Dionex, USA) and eluted with 2% acetonitrile (ACN) and 0.1% formic acid (FA) in the double distilled water at a flow rate of 3 μL/min for 5 min, and then separated on a reverse phase C18. Accucore nano-column (75 μm × 50 cm, 2.6 μm, 150 Å; Thermo Scientific, USA) by increasing buffer B from 0% to 100% within 155 min and followed with 25 min wash with 100% B at a flow rate of 300 mL/min (A: 0.1% FA in H_2_O; B: 80% ACN and 0.08% FA in H_2_O). The separated peptides were identified with a mass spectrometer (MS) (Q ExactiveTM hybrid quadrupole-orbitrap, Thermo Scientific, USA). The mass spectrometry main specifications were as follows: MS scanning range (*m*/*z*): 350–1, 300; raw data acquisition mode: high-resolution data-dependent acquisition (DDA); Top 20: the peptide molecules of Top 20 in terms of the most vigorous signal intensity in the primary mass spectrometry, which were splintered by high-energy collisional dissociation (HCD), were subject to secondary mass spectrometry analysis; polarity switching: one complete cycle in <1 s (one full positive mode scan and one full negative mode scan at a resolution setting of 60,000). Primary mass spectrometry resolution: 60,000; automatic gain control (AGC) target: 3e6; maximum injection time: 20 ms; peptide fragmentation mode: HCD. Secondary mass spectrometry resolution: 15,000; AGC target: 1e5; maximum injection time: 50 ms; fixed first mass: 100 *m*/*z*; minimum AGC target: 8e3; intensity threshold: 1.6e5; dynamic exclusion time: 18 s.

Step 4: Data processing. Proteome Discoverer™ Software 2.2 was used for proteomics analysis.

The raw files were submitted to the Proteome Discoverer server, and the database search was performed in the already-established databases. The relevant parameters of the database search are shown in Table 1S.

Step 5: Whey protein data analysis.

According to the results of mass spectrometry identification, all proteins and protein sequences were identified and compared with the major databases (KEGG, software version 2018; Pfam, software version 32; PIR ID-mapping, software version 2019; NCBI, software version 2019). The analyses of Venn, hot map, principal component analysis (PCA), differential volcanic map, and differential proteins hierarchical clustering were performed on the platform of Shanghai Major Bio-pharm Technology Co., Ltd. (https://www.majorbio.com/web/www/index).

### 2.6 Molecular docking between whey protein and lactose

The structures of whey protein *β-lactoglobulin* and *α-lactalbumin* were screened from the UniProt protein database (https://www.UniProt.org/), and further small molecules and water molecules were removed from their structures using the PyMol software. Lactose small molecule 3D structure was then downloaded from the PubChem database (https://pubchem.ncbi.nlm.nih.gov/). Auto Dock4.2 software was used to dock molecules between the two processed whey proteins and lactose, where the whey proteins *β- lactoglobulin* and *α- lactalbumin* separately act as a receptor and lactose acts as a ligand under the minimum energy mode. After the docking was completed, PyMol was used to identify the binding sites between the two whey proteins and lactose.

### 2.7 Statistical analysis

The results were expressed as means of three replicates (mean ± SD). The data were analyzed by a one-way analysis of variance (ANOVA) and were compared using Duncan's multiple range test using statistical 10.0 software (Statsoft Inc., Tulsa, OK, USA). Differences between the means were statistically significant at 0.05 (*p* < 0.05).

## 3 Results

### 3.1 SDS-PAGE and diameter of whey protein particles

**Fig. 1:**
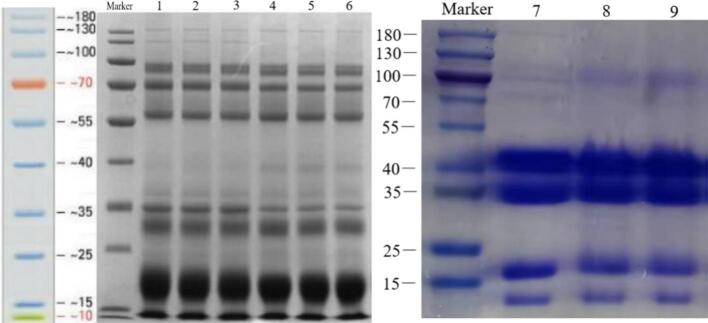
SDS-PAGE electrophoretogram of whey proteins obtained from goat milk. Note: Protein bands 1–3, 4–6, and 7–9 represent the whey proteins treated at room temperature (the control), at 135 °C(UHT) for 6 s, and at 160 °C (SDT) for 6 s, respectively.

**Fig. 2:**
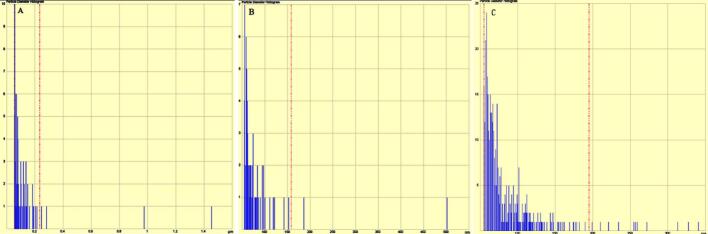
Diameter of whey protein particles obtained from MultiMode8 atomic force microscopy. Note: A, B, and C represent the control, the UHT and the SDT treatments, respectively.

**Fig. 3:**
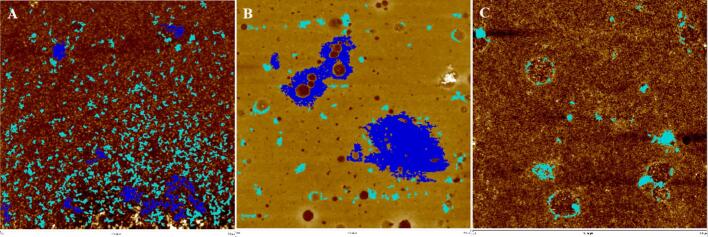
Morphology of heat-treated whey protein through MultiMode8 atomic force microscopy. Note: A, B, and C represent the control, the UHT and the SDT treatments, respectively.

SDS-PAGE electrophoretogram shows that whey protein brands following UHT treatment at 135 °C for 6 s are comparable to the control, and whey proteins subject to SDT display only four brands ranging from 10 to 45 KD, indicating significant degradation of macromolecular whey proteins due to SDT (Fig. 1). Atomic force microscopy measurements show that the diameter of whey protein particles after UHTIS treatment (50–100 μM) and SDTIS treatment (50–150 μM) increased significantly compared to the control (<0.2 μM), with particles from SDTIS treatment showing a more significant increase than those from UHTIS treatment (Fig. 2). Untreated whey protein particles were spherical and uniform. In contrast, those treated with UHT and SDT were markedly irregular and varied significantly in size (Fig. 3).

### 3.2 Concentrations of whey protein

Table 2S describes the concentrations of whey protein after various treatments. The findings showed that the concentrations of whey protein after the two high-temperature treatments were significantly lower than those of the control (*P<*0.05, *P<*0.01), and whey protein treated with UHT was considerably lower than that of the SDT treatment (*P<*0.05).

### 3.3 Venn analysis

Protein Venn analysis displays the number of proteins in each sample group and the overlapping relationships among group proteins, pinpointing common and unique proteins across groups. The Venn diagram reveals 386 proteins shared by both groups, with 16 unique proteins in the control group and 24 in the treatment group (Fig. 1S).

### 3.4 Pfam annotations of protein and differential statistical analysis

**Fig. 4:**
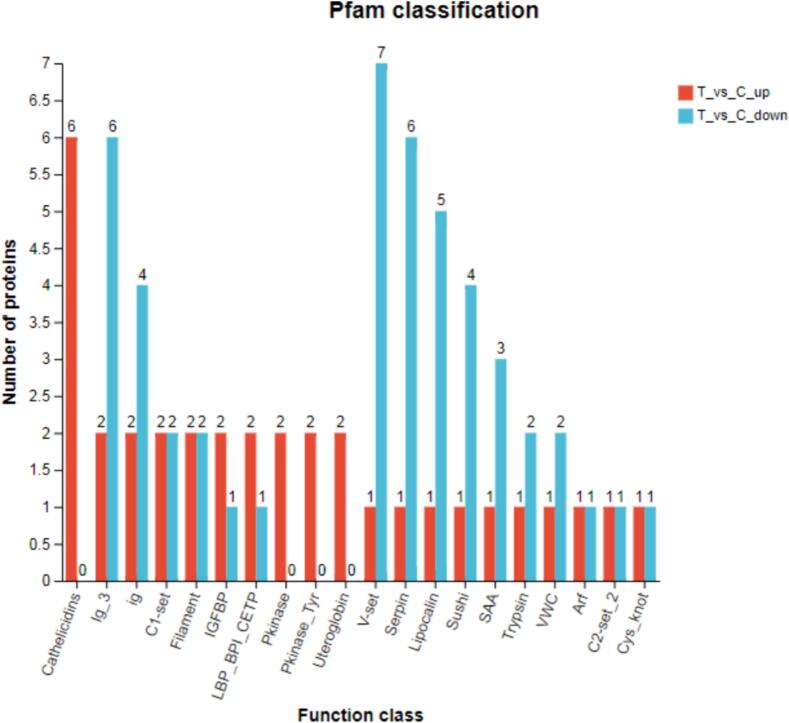
Pfam annotations of the whey proteins. Note: The horizontal axis represents the abbreviation of a functional domain, and the vertical axis represents the number of proteins annotated to each domain. C and T represents the control and UHTIS treatment, respectively.

**Fig. 5:**
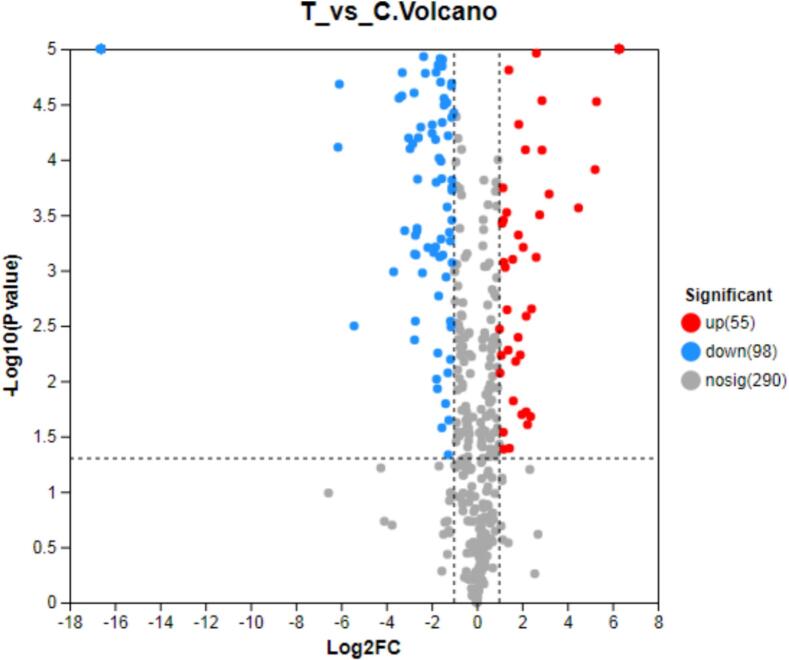
Differential statistics volcanic map. Note: The horizontal axis represents the multiplier value of the whey protein level between two samples, which is obtained by dividing the whey protein level of the treated sample by the expression level of the control sample. The vertical axis represents the statistical test value (*P* value) of the differences between the gene expression levels. The values in the horizontal and vertical axes have been logarithmized. Each point in the graph represents a specific protein. The point on the left is the down-regulated protein, opposite to the right point. Red represents the up-regulated proteins in the differential comparison group, while blue represents the down-regulated proteins. C and T represents the control and UHTIS treatment, respectively. (For interpretation of the references to color in this figure legend, the reader is referred to the web version of this article.)

In this experiment, all identified whey proteins were compared with the significant database Pfam to obtain extensive functional information and conduct a statistical analysis of the annotation status of each protein. Within the Pfam database, 1, 729 proteins were annotated through sequence alignment to functional domains, and 426 proteins in them are reliable based on the matching results. Further analysis showed that UHTIS treatment increased the presence of proteins such as cathelicidins, *kinase*, *kinase-Tyr*, *uteroglobin*, *IGFBP,* and *IBP-BPI-CETP* while decreasing proteins including Ig-3, Ig, V-set, Serpin, Lipocalin, Sushi, SAA, Trypsin, and VWC (Fig. 4). The differential volcano plot illustrates that UHTIS treatment raised the levels of 55 whey proteins and reduced the levels of 98 whey proteins compared to the control, with no effect on 290 whey proteins (Fig. 5).

### 3.5 Differential proteins hierarchical clustering

**Fig. 6:**
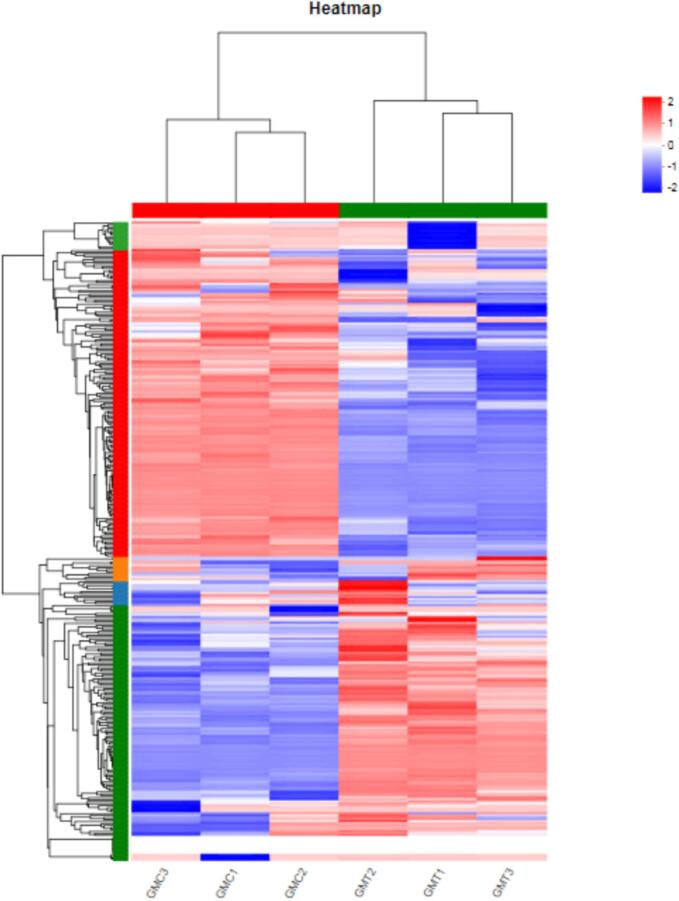
Differential proteins hierarchical clustering. Note: Each column represents a sample, and each row represents a protein. The colors represent the level of the protein in the group. Red represents a higher level of the protein in the sample, while green represents a lower expression level. The color bar in the upper left for the specific trend of the level changes of whey protein with the numerical annotation. The left side shows a tree diagram of proteins clustering, and the right side shows the specific proteins. The upper part is a tree graph of samples clustering, and the lower part is the names of the samples. GMC1–3 and GMT 1–3 represents the control and UHTIS treatments, respectively. (For interpretation of the references to color in this figure legend, the reader is referred to the web version of this article.)

The hierarchical clustering of differential proteins revealed two main categories of goat milk whey proteins based on protein levels before and after UHTIS treatment. Approximately half of the whey proteins in the treatment group exhibited an increase or decrease in content compared to the control group (Fig. 6).

### 3.6 Functional enrichment

**Fig. 7:**
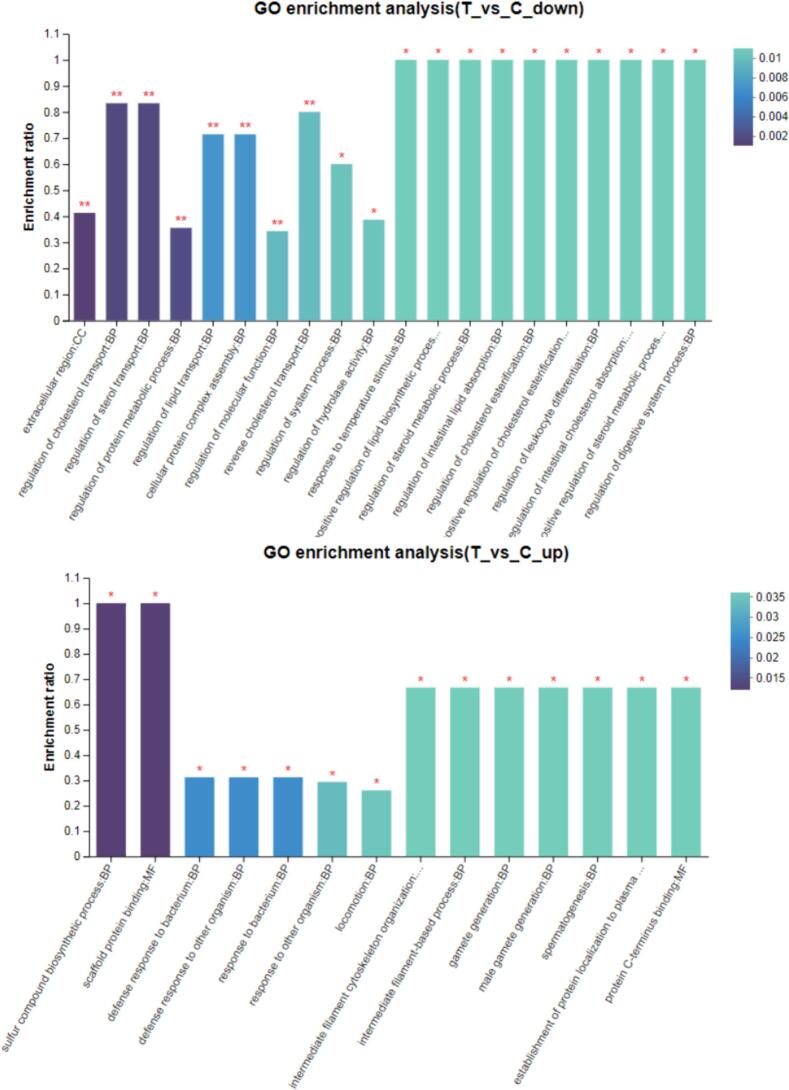
GO enrichment of functionally significant whey proteins. Note: The horizontal axis represents the GO terms, and the vertical axis represents the enrichment ratio that refers to the ratio of the protein number enriched in the GO term against the background number annotated in the GO term. The larger the ratio means the greater the degree of enrichment. The color gradient of the column on the right indicates the significance of enrichment with *P* or FDR < 0.001 marked as * * *, *P* or FDR < 0.01 marked as * *, and *P* or FDR < 0.05 marked as *. C and T represents the control and UHTIS treatment, respectively.

Functional enrichment analysis was carried out using the GO database to explore the roles of whey proteins in biological processes, cellular components, and molecular functions. The study indicated that UHTIS treatment reduced the physiological activities associated with a variety of methods, including the extracellular region, cholesterol and sterol transport regulation, protein metabolic processes, lipid transport, cellular protein complex assembly, molecular function regulation, reverse cholesterol transport, system processes, hydrolase activity regulation, temperature response, lipid biosynthesis, steroid metabolism, intestinal lipid absorption, cholesterol esterification, leukocyte differentiation, and digestive system processes (*P* < 0.05, *P* < 0.01) (Fig. 7A). Conversely, UHTIS treatment enhanced physiological activities related to sulfur compound biosynthesis, scaffold protein binding, defense responses to bacteria and other organisms, locomotion, organization of the intermediate filament cytoskeleton, gamete generation, spermatogenesis, protein localization to plasma, and protein C-terminus binding (Fig. 7B).

Regarding metabolic pathways, whey proteins treated with UHTIS may significantly upregulate pathways involved in the biosynthesis of secondary metabolites, cofactor and vitamin metabolism, and nucleotide metabolism. In contrast, the treatment notably can downregulate the metabolic pathway associated with the metabolism of terpenoids and polyketides (Fig. 2S).

### 3.7 Evaluation of carbonyl ammonia reaction between whey protein and lactose

**Fig. 8:**
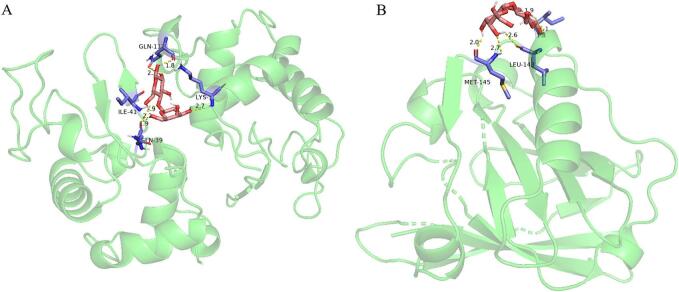
Molecular docking between whey protein and lactose. Note: A and B represent lactose molecular docking separately whey protein *β-lactoglobulin* and *α-lactalbumin.*

Heat treatment can induce protein glycosylating, known as the Maillard reaction, a carbon amide reaction between carbonyl in reducing sugars and amino compounds (amino acids and proteins). The binding structures between whey proteins and lactose were separately simulated through molecular docking software. The results showed that the amino acids docking with lactose involved Met and Leu in the whey protein *β-lactoglobulin* (Fig. 8A), and *Gln, Ile*, *Lys*, and *Gln* in the whey protein *α-lactalbu*min (Fig. 8B), indicating that Maillard reaction may occur during whey protein heat treatment.

## 4 Discussion

Milk proteins are susceptible to temperature treatments, whether above or below zero. (Regardless of whether the temperature is above or below zero). Notably, protein denaturation caused by heat treatment is more complex, including gathering, glycosylation, and hydrolysis. The present SDS-PAGE analysis indicated that SDT treatment almost completely damaged all the macromolecules in whey protein compared to the control and UHTIS treatment. This is consistent with previous findings on cheese whey from goat milk treated at 65 °C for 30 min, 80 °C for 5 min, or 90 °C for 5 min (Huang et al., 2023), also consistent with another previous research on the soluble proteins in reconstituted milk exposed to at 75 °C or 85 °C or 90 °C for 20 min (Hamouda et al., 2022; Moatsou, 2023; Zhang, Wang, Lu, Kong and Ge, 2023), suggesting that both SDT and low-temperature treatments could initiate the hydrolysis of milk proteins, meanwhile all of which could lead to the destruction of most active proteins in both whey and milk powders. Furtherly, the present work showed that treatment lowered the concentration of whey protein; this could be ascribed to the hydrolysis of the part of whey proteins.

Further, the current Venn analysis demonstrated that UHTIS treatment caused a certain degree of damage (roughly 6%) to whey protein. Still, most did not change, similar to the previous work on whey proteins (Zhang et al., 2024; Akhlaghi, 2024; Krishna et al., 2021). Meanwhile, the current work of the tree graph of samples clustering showed the same group shared with almost identical protein patterns, suggesting UHTIS treatment indeed affected the composition of whey protein, consistently with the previous works (Rosendo-Silva, Viana, Carvalho, Reis, & Matafome, 2023; Zhang, Hao, et al., 2024; Zhao et al., 2023). Meanwhile, the present work showed that the content of the whey protein marked decreased in UHTIS treatment group. So, it concluded that UHTIS treatment altered the profile of whey protein because of the hydrolysis of some whey proteins.

Though the UHTIS treatment had a light impact on the whey proteins from goat milk, it could affect some metabolic pathways human body, similar to a study on the role of proteins playing in health and disease (Wang et al., 2023; Gobena, Admassu, Kinde, & Gessese, 2024). These changes of metabolic pathways may be caused by the essential amino acids, including valine, leucine, phenylalanine, tryptophan, lysine, isoleucine, methionine, threonine, and histidine, as well as the whey proteins themselves and their degraded peptides.

The present results showed that the diameter of whey protein particles treated with UHTIS and SDTIS increased, consistent with previous works on heat-treated protein (Gobena et al., 2024; Zhao et al., 2023); this may be due to whey protein aggregation and gelation. Aggregation and gelation of globular proteins are readily induced by heating that renders the peptide chain, especially in the beta-sheets more mobile, allowing amino acids to interact with and bind to other proteins (Saguer, Alvarez, Sedman, & Ismail, 2013; Nicolai, 2019; Sabbagh, Muhamad, Niazmand, Dikshit, & Kim, 2022; Yan et al., 2023). Therefore, UHT liquid dairy products should be homogenized after heat treatment to prevent protein from precipitation during shelf-time*.*

Lactose, a unique carbohydrate in human and mammalian milk, is a disaccharide composed of glucose and galactose. Based on the reducibility of glucose, glycation of whey protein during heating should be considered (Han et al., 2024; Ozturk et al., 2024). The molecular docking results indicated that the carbonyl ammonia reaction between whey protein and lactose may occur. Dairy products subject to high-temperature treatment, especially for a long time, may produce a certain amount of protein glycosylation, which may cause allergic reactions in some human bodies (Beck et al., 2015). Consequently, the optimal combination of temperature and time for milk processing has been extensively studied to maximize the preservation of milk's inherent components, such as using high temperatures for short durations.

## 5 Conclusion

Whey protein contains many immune-active proteins and is sensitive to heat treatment. Both UHTIS and SDTIS treatments cause the whey protein particles to enlarge due to partial agglomeration, with SDT treatment notably leading to significant degradation of macromolecular whey proteins. After UHTIS treatment, Pfam annotations identified 426 proteins, including 386 common proteins and 16 unique proteins in the UHTIS-treated group and 24 in the control group, respectively. The UHTIS treatment increased the levels of 55 whey proteins and decreased 98 whey proteins, with nearly half of the whey proteins experiencing changes in concentration in the UHTIS-treated group. Additionally, UHTIS-treated whey proteins may influence three metabolic pathways positively while negatively impacting one. Overall, UHTIS treatment altered the composition of whey protein and could impact several metabolic pathways, but these changes should be further unveiled in the future. An appropriate balance between treatment time and temperature can minimize severe damage to whey protein*.* Our findings could supply approaches to develop UHT goat milk *whey protein* products that reserve more active substances, contributing to the broader aim of maximizing the nutritional benefits of dairy products.

## CRediT authorship contribution statement

**Majida Al-Wraikat:** Writing – original draft. **Mohamed Aamer Abubaker:** Writing – review & editing. **Yingli Liu:** Supervision. **Xi Ping Shen:** Methodology. **Yu He:** Methodology, Investigation. **Linqiang Li:** Project administration. **YongFeng Liu:** Supervision, Conceptualization.

## Declaration of competing interest

The authors declare that they have no known competing financial interests or personal relationships that could have appeared to influence the work reported in this paper.

## Data Availability

No data was used for the research described in the article.

## Appendix A Supplementary data

Supplementary material Image 1
