# Supplementary material for: Label-free quantitative proteomic analysis of functional changes of goat milk whey proteins subject to heat treatments of ultra-high-temperature and the common low-temperature
Source: Food Chem X. 2024 Jul 23;23:101691. doi: 10.1016/j.fochx.2024.101691 (PMC11342887; doi:10.1016/j.fochx.2024.101691)
Supplement: Supplementary file 1 — Supplementary material [file mmc1.docx]

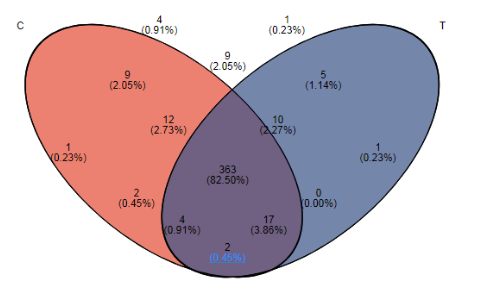


**Fig.1S.** Venn showing common and unique proteins

Note: The overlapping part in the figure represent the number of proteins shared by multiple protein sets, the non-overlapping parts represent the number of proteins unique to the protein set. C and T represents the control and UHTIS treatment, respectively.


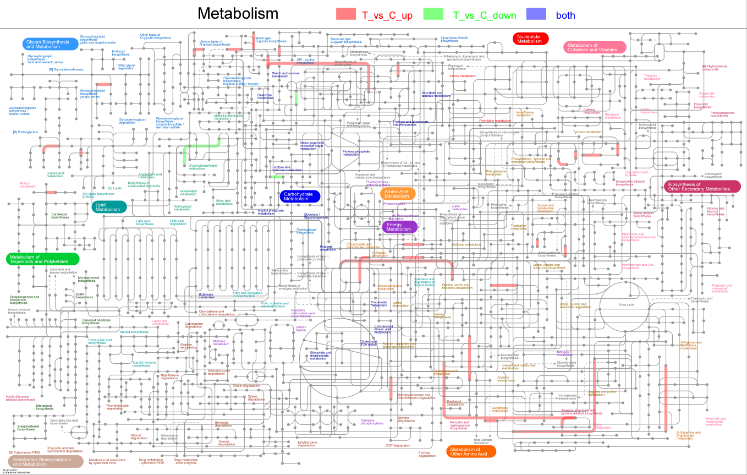


**Fig.2S**. Metabolic pathway diagram. C and T represents the control and UHTIS treatment, respectively.

**Table 1S** Proteome discoverer searching parameters

| **Item** | **Value** |
| --- | --- |
| Proteome Discoverer version | 2.1 |
| Protein Database | uniprot-mouse-79954s-20160909.fasta |
| Cys alkylation | Carbamidomethyl |
| Enzyme Name | Trypsin (Full) |
| Max. Missed Cleavage Sites | 2 |
| Precursor Mass Tolorance | 10 ppm |
| Fragment Mass Tolorance | 0.05Da |

Note: The result filtering parameter was peptide FDR ≤ 0.01.

**Table 2S** Whey protein concentrations after different heat treatments

| **Treatments** | **Concentrations (mg/mL)** |
| --- | --- |
| Control | 11.58 ± 0.73^a^ |
| UHT | 10.80 ± 0.76^b^ |
| SDT | 9.20 ± 0.74^c^ |

Note: Values with the different superscript letter are statistically different compared with the control at 0.05 or 0.01 level.
